# Impact of schooling in the HIV/AIDS prevalence among Brazilian transgender women

**DOI:** 10.20945/2359-3997000000260

**Published:** 2020-05-27

**Authors:** Rafael Loch Batista, Elisa dal Rosario Verduguez, Marlene Inacio, Flávia Siqueira Cunha, Mateus Diniz Marques, Natália Lisboa Rosa Almeida Gomes, José Antônio D Faria, Maria Helena Palma Sircili, Berenice B. Mendonça, Elaine M. Frade Costa, Sorahia Domenice

**Affiliations:** 1 Hospital de Clínicas Faculdade de Medicina Universidade de São Paulo São Paulo SP Brasil Unidade de Endocrinologia do Desenvolvimento, Laboratório de Hormônios e Genética Molecular (LIM/42), Hospital de Clínicas, Faculdade de Medicina, Universidade de São Paulo, São Paulo, SP, Brasil; 2 Departamento de Clínica Médica Universidade Federal de Santa Maria Santa Maria RS Brasil Departamento de Clínica Médica, Universidade Federal de Santa Maria (UFSM), Santa Maria, RS, Brasil

**Keywords:** Transgender women, school dropout, schooling, bullying, HIV/AIDS, LGBT

## Abstract

**Objective:**

Discrimination and bullying are common conditions among LGBT people. During schooling, these practices compromising education. The aim of this study is to evaluate educational attainment among Brazilian transgender women (TW) and how their education level affects the risk of HIV infection.

**Study design:**

a cross-sectional population-based study.

**Subjects and methods:**

95 adult TW were selected. Information concerning verbal and physical aggression, school dropout, school years (SY), and educational level were assessed. HIV status was screened using a fourth-generation immunoassay followed by western blot testing.

**Results:**

The mean of SY was 9.1 ± 3.8 ys. The mean age at school dropout was 16.3 ± 3.4 ys old. Verbal aggression was reported by 83%, physical by 48%, and 18% of the TW dropped out school immediately after being physically assaulted. Participants who suffered physical aggression attended school for almost 4 years less than those participants who did not suffer this abuse (OR = -3.96, p < 0.0001). A similar result was found for verbal aggression (OR = -4.35; p < 0.0001). HIV/AIDS prevalence was 18% (n = 17). The mean of SY among HIV/AIDS positive and negative individuals were 6.8 ± 43 versus 9.7 ± 3, respectively (p = 0.004). Lower education was associated with higher frequency of HIV/AIDS among TW and this relationship was sustained after adjustment for injectable drug use and sex work (OR = 0.79, p = 0.04).

**Conclusion:**

Among Brazilian TW, lower education level was a risk factor associated with HIV. The reasons for low schooling among TW are multifactorial, but verbal and physical harassment strongly contribute for it.

## INTRODUCTION

Discrimination, bullying, and social exclusion are common conditions among lesbian, gay, bisexual, and transgender (LGBT) people ( 1 , 2 ). Transgender individuals experience high levels of stigma-related victimization, which occurs in several settings, such as home, school, communities, work, and health institutions ( 3 ). There are many types of discrimination, such as verbal, psychological, and physical violence ( 4 , 5 ). In most cases, the insults come from their cisgender peers ( 3 ).

In the United States, 92.3% of transgenders suffer some form of verbal aggression ( 6 ), while bullying was reported by 89.5% of American transgender people ( 4 , 7 ). Bullying also impacts on schooling because LGBT people have higher rates of school absenteeism (often related to fears of being bullied at school) and school dropout ( 8 - 11 ). Low schooling is clearly related to low income, poverty, and unemployment, affecting negatively the quality of life.

Bullying plays a role in several risk behaviors among LGBT youth and has been associated with higher addiction to various substances, including tobacco, alcohol and methamphetamines ( 12 - 14 ). It has been estimated that LGBT youth who are bullied may engage in risky sexual behaviors, such as having unprotected sex ( 11 , 15 ), which may collaborate to alarming burden of HIV in transgender women (TW) ( 16 - 18 ).

## SUBJECTS AND METHODS

A cross-sectional population-based study was conducted with 95 adult TW (mean age 36, ranging from 21 to 68 years) followed at Gender Dysphoria Unit of the *Hospital das Clínicas da Faculdade de Medicina da Universidade de São Paulo* (HCFMUSP). All participants had gender dysphoria, according to the Diagnostic and Statistical Manual of Mental Disorders (DSM-V)/International Classification of Diseases (ICD-10).

Data regarding schooling, economic status, and violence presence (verbal, physical and sexual) were assessed.

Household monthly income was categorized as low income (less than R$ 1,000 Brazilian reals, which is equivalent to 250 American dollars by each person in the same house or more than R$ 1,000).

HIV status was defined using an algorithm starting with a fourth-generation immunoassay for screening followed by western blotting test in positive cases.

The local Ethics Committee of the HC-FMUSP approved this study and a signed informed consent form was obtained from all participants. This study was also approved by Plataforma Brasil under the number CAAE: 17986914.3.0000.0068.

### Statistical analysis

Numerical variables were presented as mean ± SD. Categorical variables were analyzed using a chi-squared test. For continuous variables, t-test was used for variables with normal distribution and in case of non-normality a Mann-Whitney U test was used. Odds ratios and corresponding 95% confidence intervals for HIV/AIDS prevalence was estimated using logistic regression. The impact of verbal and physical aggression in school years was estimated by univariate analysis. Logistic regression was applied to estimate independence of each variable on HIV risk through 3 models: model 1 was the univariate analysis; model 2 was adjusted for school years, prostitution, and use of injectable substances; model 3 was adjusted to sex work, use of injectable drugs, physical aggression, and verbal aggression. P values of 0.05 or less were considered to indicate statistical significance. All statistical analysis were performed using statistical software Stata/SE 15.0 (StataCorp LLC, Texas, USA).

## RESULTS

In this cohort of Brazilian TW, the mean age was 33.7 ± 7.28 at the time of the study. The mean age of start at school was 7.2 ± 1.1 years old and the mean school years were 9.1 ± 3.8. The mean age for school dropout was 16.3 ± 3.4 years old. Only four participants (4.2%) had a college degree. Most participants (78%) reported that the main reason for dropping out of school was bullying by peers and harassment by school staff. Verbal aggression was reported by 83% (n = 79) of the participants and physical aggression by 48% (n = 46). Eighteen percent (n = 17) dropped out of school immediately after experiencing physical aggression. Other factors contributing to school dropout were identified as economic barriers (13%), lack of educational interest (2%), and choosing to work in a family business (2%).

Participants who suffered physical aggression attended school for almost 4 years less than those participants who did not suffer abuse. The odds ratio (OR) for physical aggression impacts on schooling was -3.96 (CI 95% -5.34 to -2.59; p < 0.0001) and the OR was similar (OR = -4.35; CI 95% -6.38 to -2.31; p < 0.0001) for those who suffer verbal aggression.

Regarding to jobs and occupations, most of the Brazilian TW had positions related to “personal services” (34.7%), domestic (22.4%), and general services (18.3%). Technical training was not required in 79.5% of these jobs. Sixty percent of TW evaluated were employed and 8% referred to themselves as sex workers.

Considering personal income, there was a significant positive correlation between school years (R = 0.506; p = 0.03) and personal income, reinforcing the concept of a close correlation between education levels and income. The rates of unemployment (p = 0.01), informal job (p = 0.02), and low personal income (p = 0.01) were significantly worse in people with lower schooling.

**Figure 1:**
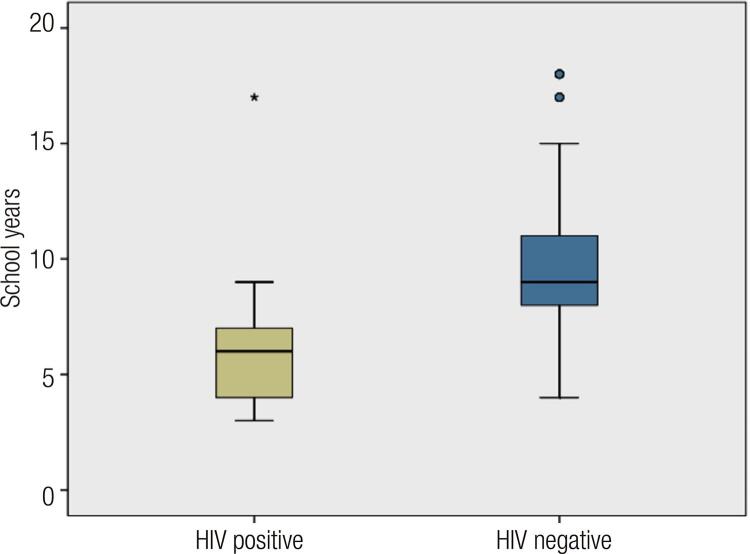
Box plot of school years in HIV positive and HIV negative.

The prevalence of HIV/AIDS in this cohort was 18% (n = 17). The mean school years among HIV/AIDS positive and negative TW individuals was 6.8 ± 4.0 and 9.7 ± 3.6 years, respectively (p = 0.004) ( Figure 1 ). In the univariate analysis, there was a risk for HIV due to low household monthly income (OR = 12.8; p < 0.001), injectable drug use (OR = 12.8; p < 0.001), and sex work (OR = 6.36; p = 0.001).

Reduced schooling was associated with higher frequency of HIV/AIDS diagnosis after adjustment for injectable drug use and prostitution, with an OR = 0.79 (CI 95% 0.63 to 0.99, p = 0.04) ( Table 2 ).

**Table 2:** Univariate analysis of impact of verbal and physical aggression in school years

	Coef	P	CI 95%
Physical aggression	-3.96	<0.001	-5.34 to -2.59
Verbal aggression	-4.35	<0.001	-6.38 to -2.31

**Table 3:** Logistic regression for impact of school years in risk for HIV

	OR	P	CI 95%
Model 1	0.73	0.007	(0.58-0.92)
Model 2	0.79	0.04	(0.63-0.98)
Model 3	0.74	0.02	(0.57-0.96)

Model 1 was univariate analysis (HIV vs school years). Model 2 was adjusted to sex work and use of injectable drugs and Model 3 was adjusted to sex work, use of injectable drugs, physical aggression and verbal aggression.

## DISCUSSION

Many transgender students experience discrimination and harassment at college, which may have implications for their academic success and retention. A national American survey, with nearly 6,500 transgender people, found that 35% of them reported negative treatment by other students, teachers, and staff. In another survey, 24% of 27,000 transgender individuals reported being verbally, physically or sexually harassed at school. Not surprisingly, 75% of trans students report feeling insecure at school. The consequence is school dropout: it has been estimated that 1 in 6 trans leaves school before completing their studies and 16% leave school immediately after an assault (9- 11 ).

Few data are known about educational profile of transgender population in Brazil, but also among Brazilian transgender youth, the major reason for school dropout is bullying. This condition was confirmed in our study, in which 78% of TW appointed bullying as the main reason for school dropout. Verbal aggression was the most common form of harassment (83%; n = 79) and physical aggression was also frequent (50%, n = 48). Both caused similar impact on schooling, reducing school years by 4.

The mean of school years observed in this TW cohort was 9.1 years, similarly to the data reported in a TW cohort from the South of Brazil (10 school years) ( 19 ). In the same way, the frequency of TW with superior education in these two Brazilian TM cohorts was 4.2%. This index was very low considering that 23% of Brazilian cis women had superior education (Ministério da Educação, www.portal.mec.gov.br, accessed in May 25, 2019).

Research based on developing world has identified educational status as a major predictor of health outcomes and economic trends in the industrialized world have intensified the relationship between education and health ( 20 - 22 ). The reasons why education is so impactful on health is still not clear, but several pathways to health outcomes may be indirect, by attainment of better socioeconomic circumstances or behavior, potentially including health behaviors.

Employment limitations can push trans people towards informal jobs that have low potential for personal growth and development, such as beauticians, entertainers, domestic services or sex workers, as also observed in our group of TW ( 23 , 24 ). The rate of unemployment among the members of this Brazilian TW cohort was 40%. A high hate of unemployment is also reported in other transgender’s cohorts worldwide. ( 17 , 23 , 24 ). However, comparing with the Brazilian cis population, the rate of unemployment in 2018 was 13.3%, which is lower than that observed among the TW evaluated in this study.

The real prevalence of prostitution in transgender people is difficult to be straightly estimated, but the prevalence of HIV/AIDS among transgenders who are sex workers is very high, ranging from 24% to 75% ( 24 , 25 , 26 ). In the present study, schooling among sex worker participants was significantly lower than in individuals with other jobs. It may suggest that the frequency of the sex workers among the TW reflects their socioeconomic disadvantage and a high risk to HIV/AIDS infection.

The frequency of injectable illicit drug use among positive HIV/AIDS TW was significantly higher than in negative HIV TW in our cohort ( Table 1 ). The abuse of illicit substances among LGBT people has been documented and associated with high frequency of depressive and anxious disorders ( 7 ). In transgender individuals, due to socioeconomic disadvantages, sex work can be a way to finance drug use. Additionally, the prevalence of illicit drug use among sex worker transgenders is high ( 12 , 16 ).

**Table 1:** Odds ratio for HIV in male-to-female Brazilian transgenders

	HIV+	HIV-	CI	P	OR
School years*	6.8 (±4.0)	9.6 (±3.6)		0.004	
Household monthly income					
<1,000	15	21	3.7-4.91	<0.001	12.8
>1,000	3	54			
Sex work					
Yes	7	7	1.8-2.67	0.001	6.36
No	11	70			
Employed					
Yes	16	57		0.23	
No	2	18			
Injectable drug use					
Yes	5	4	1.7-31.9	0.02	7.5
No	12	72			

Although a lot of improvement has been achieved in health care for people with HIV/AIDS, HIV infection disproportionately affects people who are often highly marginalized and stigmatized, including transgender people. HIV/AIDS frequency in different transgender cohorts has been estimated around 19% ( 25 ), which is very similar to that identified in our study (18%) and extremely higher than the estimated prevalence for general Brazilian population (0.6%) (OR = 55.55, CI 95% 38.39 to 80.39) ( 19 ). This difference can be explained by several factors as risky sexual behavior, anal intercourse, multiple sexual partners, sex work, and injectable drug abuse. However, other aspects play a role to increase HIV/AIDS frequency among TW. According to our results, low schooling was related with HIV in an independent way, regardless sex work, previous physical and verbal harassment, and illicit injectable substances use.

As HIV remains a big issue for transgender people at epidemic levels, any action should be taken, either to reduce new cases or to hold HIV epidemy. Control of HIV should be based on understanding of local patterns, which include local HIV prevalence and incidence, epidemic and risk factors among key populations. HIV primary prevention by use of oral pre-exposure prophylaxis (PrEP) has shown consistent results in HIV prevention in transgender and non-transgender people ( 27 , 28 ). However, the effect of PrEP depends on uptake, sexual practices, and adherence ( 26 ). In a large recent cohort, testing PrEP effectiveness in HIV primary prevention on transgender and non-transgender people, adherence to PrEP strategy was higher in individuals with more schooling years ( 26 ). It suggests that lower schooling can be a barrier for effective HIV prevention by PrEP strategy.

### Limitations

The population of this study came from a single tertiary medical center in Brazil and our findings may be not generalized for TW in other regions of Brazil, other social conditions or cultures. Some data were self-reported, therefore some information could be missed/omitted by the participants. Finally, our sample size was limited to the patients with regular follow-up in the outpatient ambulatory of HCFMUSP. Large TW cohorts followed for a long time may contribute to clarify other determinants for HIV infection in this population.

In conclusion, there is a high prevalence of HIV/AIDS among this Brazilian cohort of transgender women. In this population, lower schooling was an independent risk factor associated with HIV infection. The reasons for low schooling among transgender people are multifactorial, but verbal and physical harassment strongly contribute for school dropout and low schooling. Strategies to avoid scholar bullying and harassment would be helpful to reduce scholar dropout, to increase schooling and to improve health for transgender women.
